# Design Principles for Ligand-Sensing, Conformation-Switching Ribozymes

**DOI:** 10.1371/journal.pcbi.1000620

**Published:** 2009-12-24

**Authors:** Xi Chen, Andrew D. Ellington

**Affiliations:** Department of Chemistry and Biochemistry, Center for Systems and Synthetic Biology, University of Texas at Austin, Austin, Texas, United States of America; University of Michigan, United States of America

## Abstract

Nucleic acid sensor elements are proving increasingly useful in biotechnology and biomedical applications. A number of ligand-sensing, conformational-switching ribozymes (also known as allosteric ribozymes or aptazymes) have been generated by some combination of directed evolution or rational design. Such sensor elements typically fuse a molecular recognition domain (aptamer) with a catalytic signal generator (ribozyme). Although the rational design of aptazymes has begun to be explored, the relationships between the thermodynamics of aptazyme conformational changes and aptazyme performance *in vitro* and *in vivo* have not been examined in a quantitative framework. We have therefore developed a quantitative and predictive model for aptazymes as biosensors *in vitro* and as riboswitches *in vivo*. In the process, we have identified key relationships (or dimensionless parameters) that dictate aptazyme performance, and in consequence, established equations for precisely engineering aptazyme function. In particular, our analysis quantifies the intrinsic trade-off between ligand sensitivity and the dynamic range of activity. We were also able to determine how *in vivo* parameters, such as mRNA degradation rates, impact the design and function of aptazymes when used as riboswitches. Using this theoretical framework we were able to achieve quantitative agreement between our models and published data. In consequence, we are able to suggest experimental guidelines for quantitatively predicting the performance of aptazyme-based riboswitches. By identifying factors that limit the performance of previously published systems we were able to generate immediately testable hypotheses for their improvement. The robust theoretical framework and identified optimization parameters should now enable the precision design of aptazymes for biotechnological and clinical applications.

## Author Summary

Aptamers are nucleic acids that bind their cognate ligands (ranging from metal ions to small molecules to proteins) specifically and tightly. Through rational design and/or directed evolution, aptamers can be engineered into allosteric nucleic acids whose conformations can be regulated by their ligands. Aptamer beacons, aptazymes, and riboswitches all undergo ligand-dependent conformational changes, and have been adapted to signal the concentration of their ligands. However, there is currently no model that can be used to predict how the energetics of conformational change affects signaling, either *in vitro* or *in vivo*. We have developed a model that identifies what parameters can be optimized to best yield signals. By focusing on these parameters, it should be possible to more readily design or select more effective conformation-switching nucleic acid biosensors.

## Introduction

Nucleic acid binding species (aptamers) have emerged as a powerful tool for molecular recognition, and have begun to be widely adapted as biosensors, in drug-delivery systems, and as regulatory elements that control gene expression [1]–[4]. Naturally occurring nucleic acid regulatory elements, riboswitches, have been discovered in a variety of organisms and control the expression of a wide range of genes [5].

One of the major advantages of aptamers over their protein counterparts is that they can be easily coupled to other functional RNAs based largely on secondary structural considerations in order to generate allosteric constructs. To a large extent aptamer-based biosensors (both *in vitro* and *in vivo*) can be classified into two major categories: (i) those in which the aptamer binding influences the hybridization state of other nucleic acids (for *in vitro* examples see [6],[7]; for *in vivo* examples, see [8]), and (ii) those in which aptamer binding influences the catalysis of a ribozyme (for *in vitro* examples, see [9]–[11]; for *in vivo* examples, see [12]–[15]. These allosteric ribozymes derived from aptamers are also known as aptazymes.

While there are numerous empirical examples of aptazymes operating as biosensors and regulatory elements, quantitative analyses of aptazyme performance and the development of design principles for aptazymes have seldom been attempted and are largely incomplete [10],[16]. Recently, Beisel and Smolke developed a similar model for riboswitch function [16]. However, only qualitative trends were reported. For example, while it was concluded that “a design that is biased toward forming the disrupted-aptamer conformation will generally increase the dynamic range …(but) require higher ligand concentrations to modulate protein level,” the more useful quantitative relationship between dynamic range of activity and ligand sensitivity that should enable rational design was not described. Similarly, the impact of fundamental kinetic parameters such as the ribozyme cleavage rate constant and mRNA degradation rate constant on the behavior of riboswitches was not analyzed. Additionally, those numerical solutions that were given were based on arbitrary parameters. For all of these reasons it is unclear what parameters need to be measured for the quantitative prediction of riboswitch function. It is also unclear how and to what extent the parameters can be optimized for improved function.

To establish a better quantitative understanding of aptazyme-based biosensors and riboswitches, we analyze a two-state model for aptazyme function and illustrate: (i) the quantitative relationship between the dynamic range of activity and ligand sensitivity; (ii) the variables that limit aptazyme function; (iii) the minimal set of readily measurable parameters that are necessary and sufficient to quantitatively predict aptazyme function; and (iv) strategies to design optimal aptazyme-based biosensors for both *in vitro* and *in vivo* applications. In addition, we apply this model to published data for a previously engineered riboswitch system [14] and show that this system is severely limited both by slow ribozyme cleavage relative to mRNA degradation and likely by the intracellular concentration of theophylline.

## Results/Discussion

### Schemes for the design of aptazymes

**Figure 1:**
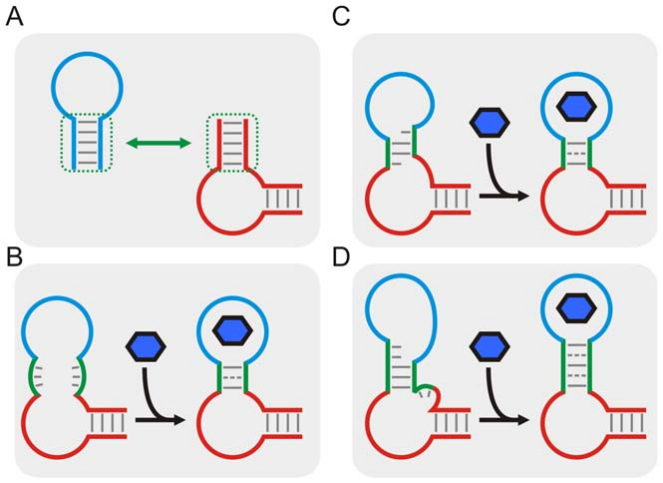
Schemas for aptazyme design. (A) The general strategy for designing aptazymes, where the aptamer and the ribozyme are shown in blue and red, respectively. The stem used to connect the aptamer and the ribozyme (the communication module) is highlighted in a dotted green box. (B) Schema for ‘binding-assisted stem-formation.’ (C) Schema for a ‘slip structure.’ (D) Schema for ‘strand replacement’. In (B) to (D), the aptamer domain, the ribozyme domain and the communication module are shown in blue, red and green, respectively. The ligand for the aptamer domain is shown as a blue hexagon. Long gray lines indicate base-pairing; short grays lines (on the left) indicate un-paired bases; and dashed gray lines (on the right) indicate mis-paired bases or non-canonical base-pairs.

The ability to predict the secondary structure of functional RNA molecules has made it possible to rationally design allosteric ribozymes. Aptamer secondary structures are superimposed upon or swapped with portions of ribozyme secondary structures (**Figure 1A**), and interactions between the two domains are often controlled by junction sequences (so-called communication modules). One commonly used strategy to design ligand-activated aptazymes can be described as ‘binding assisted stem-formation’ (**Figure 1B**) in which a weak but functionally important stem that is shared by the aptamer and the ribozyme is stabilized by ligand-binding [12],[13]. Other design strategies include ‘slip structures’ (**Figure 1C**; [9]) and ‘strand replacement’ (**Figure 1D**; [14],[15]). In these latter strategies the ligand-induced stabilization of the aptamer helix causes a conformational change in the secondary structure of the ribozyme that either promotes or inhibits catalysis. Taken together, all of these strategies assume a two-state model for the aptazyme in which one of the states is stabilized by ligand-binding.

To garner better insights into how to design aptazymes, we will attempt to model the interrelationships between aptazyme conformational change, ligand-binding, and catalysis. In this way we can separate *intrinsic* variables (including the aptamer∶ligand affinity and the ribozyme catalytic rate constant) from *extrinsic* or ‘engineerable’ variables (including the equilibrium constant between the two conformers). While the catalytic rates of the less active conformer and the more active conformer are also extrinsic variables, they should almost always be minimized (to zero if possible) and maximized (to the rate of the ribozyme sans aptamer if possible), respectively. For simplicity, we develop our analyses with self-cleaving aptazymes, but the model should be generalizable to aptazymes with other catalytic activities.

### A model for ligand-activated and ligand-inhibited aptazymes as *in vitro* biosensors

**Figure 2:**
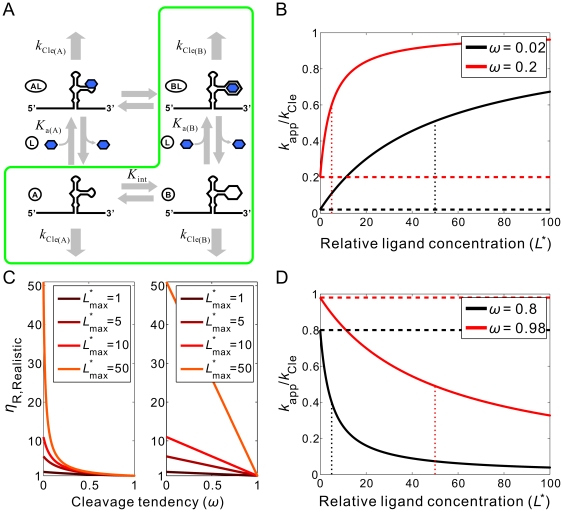
Kinetic model and performance of aptazymes as *in vitro* biosensors. (A) Two-state model for aptazyme function. The aptazyme conformers with low and high affinities for ligand are shown as *A* and *B*, respectively. The ligand-bound states of these two conformers are shown as *AL* and *BL*. *K*_int_ is the equilibrium constant for the *A*-to-*B* transition. *K*_a(A)_ and *K*_a(B)_ are the association constants for the ligand (*L*, shown as a blue hexgon) with the *A* conformer and the *B* conformer, respectively. The first-order cleavage rate constants for conformer A and conformer B are defined as *k*_Cle(A)_ and *k*_Cle(B)_, respectively. Under certain conditions (see text), the *AL* conformer can be ignored and the model can be reduced to the enclosed green box. (B) The effect of cleavage tendency (*ω*) on the performance of ligand-activated aptazymes. (D) The effect of cleavage tendency (*ω*) on the performance of ligand-inhibited aptazymes. In (B) and (D) the relative ligand concentration ([*L*_tot_]/*K*_d_) is shown on the horizontal axis and the relative apparent cleavage rate constant (*k*_app_/*k*_Cle_) is shown on the vertical axis. The basal cleavage rate constants in the absence of ligand are shown by horizontal dashed lines. The values of 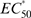 are shown as a vertical dotted lines. (C) The relationship between the cleavage tendency (*ω*) of an aptazyme and the realistic ligand-dependent change in activity (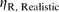) is shown for ligand-activated aptazymes (left) and ligand-inhibited aptazymes (right). This relationship is shown for different maximum available ligand concentrations (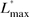).

The general model for ligand-modulated ribozymes is similar to that for allosteric protein enzymes (**Figure 2A**). In this model, the aptazyme can assume two interchangeable conformations *A* and *B* with internal equilibrium constant *K*_int_ (see **Text S3** for a summary of terms), each of which has particular (but different) ligand-binding affinities defined by association constants *K*_a(A)_ and *K*_a(B)_, respectively, and particular (but different) cleavage activities defined by cleavage rate constants *k*_Cle(A)_ and *k*_Cle(B)_, respectively. Since in most cases it is the local structure of the catalytic core (as opposed to the ligand-binding site) that determines the catalytic activity of the aptazyme, it is assumed that the aptazyme-ligand complexes *AL* and *BL* have the same cleavage rate constants as the unbound aptazymes *A* and *B*, respectively. Furthermore, we only consider the situation where all four species (*A*, *B*, *AL* and *BL*) are in equilibrium at the start of the reaction. When the conformer that possesses higher ligand-binding affinity also has higher catalytic activity the aptazyme is called ligand-activated; when the conformer that possesses higher binding affinity has lower catalytic activity the aptazyme is called ligand-inhibited. In general we assign conformation *B* to have the higher ligand-binding affinity (*K*_a(B)_>*K*_a(A)_). Thus, ligand can be thought to thermodynamically shift the *A* conformer towards *B*. Since most two-state models for allosterism assume that the ligand primarily influences the population of catalytically inactive and active conformations, we assume that the less catalytically active conformer has zero activity and the more catalytically active conformer has the same cleavage rate constant as the ribozyme sans aptamer (denoted as *k*_Cle_). Formally,
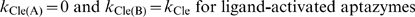
and
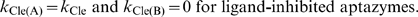



Another simplifying assumption is that the complex *BL* is much more thermodynamically stable than *AL*, and thus we can ignore the existence of *AL* and reduce the model to the path outlined in green in **Figure 2A**. This reduced model assumes that the *A* conformer must spontaneously refold into the *B* conformer in order to bind the ligand and thus excludes ligand-induced refolding of the aptazyme. This reduction is valid when two conditions are met: (i) the energy barrier between *A* and *B* is not much higher than that between *AL* and *BL*, so that aptazyme refolding does not rely on the ligand as a catalyst; and (ii) when the aptazyme is bound to the ligand the aptazyme almost exclusively assumes the *BL* conformation. We will use this reduced model in the following analyses.

The *in vitro* performance of a self-cleaving aptazyme is usually evaluated by plotting the first-order apparent cleavage rate constant (*k*_app_; the initial cleavage rate divided by the total concentration of aptazyme) against the total ligand concentration ([*L*_tot_]). As a starting point of our model, we show how *k*_app_, which is in fact contributed to by all three aptazyme conformations, is determined by the variables shown in **Figure 2A**.

Assuming that ligand-binding is much faster than aptazyme cleavage ([*L*]*k*_on(B)_ + *k*_off(B)_≫*k*_Cle(B)_) the initial cleavage rate constant should directly reflect the initial fraction of each of the three conformers *A*, *B*, and *BL*:
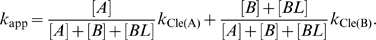
(1)If ligand-binding is slow relative to cleavage, the apparent rate constant would reflect the rate of binding (the rate limiting step) instead of cleavage. Based on the assigned definitions for parameters (see **Text S1** for derivation) the fraction of *A* can be calculated to be:
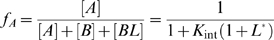
(2)and the total fractions of *B* and *BL* are:
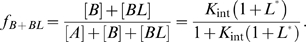
(3)where 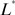 is relative ligand concentration, defined as the ligand concentration divided by the dissociation constant (*K*_d_ or 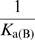) of the aptamer domain. The introduction of relative ligand concentration means that *K*_d_ is only a scaling factor for ligand concentration. In other words, two aptazymes with the same *K*_int_ but different *K*_d_ values would be indistinguishable in terms of their performance with respect to relative ligand concentrations.

In the absence of ligand, *f_B+BL_* and *f_A_* equal to 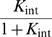 and 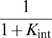, respectively. Thus the ratios 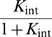 and 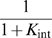 are the fraction of cleavage-competent conformers in the absence of ligand for ligand-activated aptazymes and ligand-inhibited aptazymes, respectively. We term these ratios ‘*cleavage tendency*’ and denote them as *ω*. Formally:
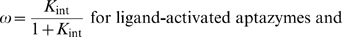


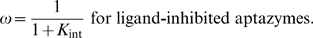



It should be noted that the relationship between *ω* and *K*_int_ is dependent on the type of the aptazyme (ligand-activated or ligand-inhibited). When the aptazyme type is specified, *ω* can be used interchangeably with *K*_int_. Since in many cases the equations are in simpler form when *ω* is used instead of *K*_int_, we will primarily use *ω* in the following derivations and analyses. From equations (1∼3) and earlier assumptions, the relationship between *k*_app_ and the relative ligand concentration 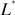 are:
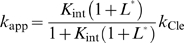
or
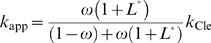
(4)for ligand-activated aptazymes, and:
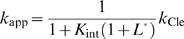
or
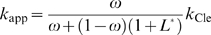
(5)for ligand-inhibited aptazymes.

### Design principles for ligand-activated aptazymes as *in vitro* biosensors

The *k*_app_-vs-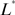 curve is an increasing hyperbola for ligand-activated aptazymes. The relationship between the parameters that describe the hyperbola (highest value, lowest value, and half-value concentration) and the model parameters (*ω* and *k*_Cle_) can be determined by rewriting equation (4) as:
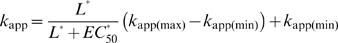
where *k*_app(min)_ and *k*_app(max)_ are the minimal and maximum apparent cleavage rate constants. These rate constants are reached in the absence of ligand and at a saturating concentration of ligand, respectively. 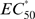 is the relative ligand concentration at which the *k*_app_ is half-way between *k*_app(min)_ and *k*_app(max)_. As a result:
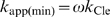
(6)

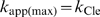
(7)

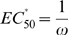
(8)


According to the definition of relative concentration, 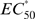 is dimensionless and scales relative to the *K*_d_ of the aptamer domain, the absolute 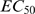 (with unit of a concentration) can be calculated with the equation:
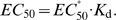
It is noteworthy that *EC*_50_ is often regarded as the ‘apparent *K*_d_ of the aptazyme’ and can be confused with *K*_d_. In fact, the *K*_d_ is an *intrinsic* variable reflecting the affinity between the aptamer and the ligand, while *EC*_50_ is design-dependent. From equation (8) it can be seen that 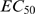 is always greater than *K*_d_ and is inversely correlated with *ω*, since the ligand binding-competent conformation *B* is only a fraction of the total aptazyme population and a smaller *ω* means this conformation is proportionately disfavored.

In addition to 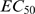, another important parameter for describing the performance of a ligand-activated aptazyme is the fold-activation of the cleavage rate constant when ligand concentration increases from 0 to infinite. We denote this fold-activation as 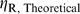 which is defined as:
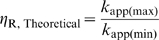
Comparing equations (6) and (7) it is obvious that for ligand-activated aptazymes,
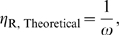
(9)which means that the maximum fold-activation is solely determined by the cleavage tendency of the aptazymes. In order to engineer aptazyme that have a higher 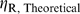, one must minimize *ω*, i.e. the cleavage-competent conformation should be disfavored in the absence of ligand. For example to achieve a >10^2^-fold activation in the presence of ligan *ω* should also be no greater than 10^−2^, which in turn means that the free energy of conformation *A* should be disfavored by at least 2.8 kcal/mole (at 37°C) relative to conformation *B*. However, a low value of *ω* would also increase the concentration of ligand that was required to fully activate the aptazyme. This can be seen by comparing equations (8) and (9), yielding:
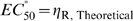
or
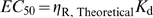
(10)


In other words, high sensitivity (low *EC*_50_) and a large dynamic range of *k*_app_ (high 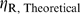) cannot be obtained simultaneously (**Figure 2B**). Conversely, if an aptazyme displays a mediocre 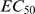 and also has a large fold-activation it can be inferred that the aptamer domain may actually have a very high affinity for its ligand. For example, a lysozyme-dependent L1-ligase previously selected by Robertson and Ellington [11] exhibits an *EC*_50_ of 1.5 µM but has a 3100-fold activation in the presence of saturating concentration of ligand (which means 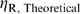
≥3100). According to equation (10), the aptamer domain of this aptazyme may have a *K*_d_ as low as 500 pM.

To reach the full theoretical dynamic range of *k*_app_, the ligand concentration should vary from 0 to infinite, which is of course impossible. The upper limit of the *realistic* dynamic range of *k*_app_ for a ligand-activated aptazyme is determined by the *k*_app_ at the highest possible concentration of ligand. Therefore, when designing aptazymes it is important to consider the fold-activation of the cleavage rate constant when ligand concentration increases from 0 to its highest possible concentration. We denote this fold-activation as 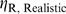 and formally define it as:
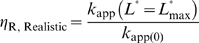
where the 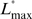 is the highest possible relative ligand concentration.

Since decreasing cleavage tendency is a double-edged sword in that it increases 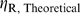 but at the same time requires higher ligand concentration to achieve half activation, it is important to find the cleavage tendency that gives optimal aptazyme performance (the highest 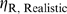). To find the optimal cleavage tendency, it is useful to determine the explicit expression of 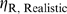 as a function of *ω*, which is:
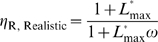
(11)Interestingly, from this equation it is clear that for any 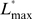
>0, 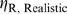 increases monotonically as the cleavage tendency *ω* decreases, as shown in (**Figure 2C** **, left panel**). In other words, it is always beneficial to have a lower cleavage tendency when the goal is to design the aptazyme to maximize 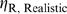.

Practically, the only negative effect of engineering small cleavage tendencies in aptazymes is that the absolute value of 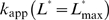 is small, and thus the rate of cleavage and signal generated by the aptazyme may be small. Therefore, as a practical guideline for designing ligand-activated aptazymes as *in vitro* biosensors the cleavage tendency should be minimized as long as the value 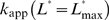 still falls within a range that is readily detected by a given assay.

### Design principles for ligand-inhibited aptazymes as *in vitro* biosensors

The *k*_app_-vs-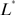 curve for a ligand-inhibited aptazyme is a decreasing hyperbola, whose descriptor can be solved by rearranging equation (5) to the form:
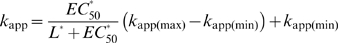
yielding:
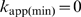
(12)

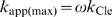
(13)

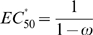
(14)


Here the definition of the fold-inhibition over the theoretical dynamic range of *k*_app_ (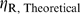) is problematic since the theoretical lower limit of *k*_app_ is 0 and therefore 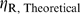 for a ligand-inhibited aptazyme would be infinite. The value 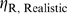 (now defined as 
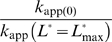
) will be dependent on the design of the aptazyme (i.e., the choice of the cleavage tendency *ω*) and on the highest available concentration of ligand (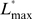). Because the inhibited aptazyme is hyperbolically controlled by the ligand (see **Figure 2D**), the lower realistic limit of *k*_app_ will be very hard to reach, and the range of *k*_app_ values for ligand-inhibited aptazymes will be heavily dependent on the ratio of 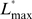 to 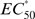. A low 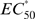 will be crucial if the highest possible concentration of ligand is limited or if the intrinsic affinity of the aptamer domain is low.

According to equation (14), a lower 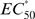 should be engineered by decreasing *ω*. However, by comparing equations (13) and (14) we find:
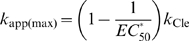
(15)which in turn implies that lowering 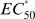 will decrease the upper bound on possible *k*_app_ values (**Figure 2D**). Once again there is a compromise between ligand sensitivity and the dynamic range of activity.

Again, to find the cleavage tendency that yields the highest 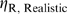 for a given 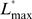, the expression of 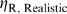 as a function of *ω* should be considered. This expression is:
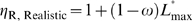
(16)Interestingly, as cleavage tendency *ω* increases from 0 to 1, 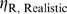 decreases linearly from 1+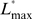 to 1 (**Figure 2C** **, right panel**). Consequently, when designing ligand-inhibited aptazymes as *in vitro* biosensors, it is also always beneficial to choose a low cleavage tendency as long as 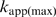 is still readily detectable.

In summary, for both ligand-activated and ligand-inhibited aptazymes there are trade-offs between ligand sensitivity and the dynamic range of activity, reflected by equations (10) and (15), respectively. However, when attempting to maximize 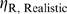 it is always a good strategy to choose a low cleavage tendency, as shown by equations (11) and (16) and **Figure 2C**.

### Aptazymes as *in vivo* riboswitches

Aptazymes can be inserted into mRNAs in order to regulate their stabilities and translation efficiencies, thereby functioning similar to natural riboswitches *in vivo*. In such applications, aptazyme regulation will of necessity be further modulated by the dynamic processes surrounding RNA metabolism, including transcription, processing, transportation, translation and degradation. In addition, the most readily observed signals will be steady state mRNA or protein concentrations, instead of cleavage rate constants.

The most straightforward strategy for adapting aptazymes to gene regulation is to engineer a drug-responsive cleavase (such as a hammerhead aptazyme) to target a particular mRNA. However, despite decades of effort, gene regulation based on *trans*-cleaving ribozymes has proven largely unsuccessful. Gene regulation via ligand-responsive ribozyme was paradoxically first demonstrated in a natural system, where a novel ribozyme located at the 5′ UTR of the *glmS* gene of *B. subtilis* was found to self-cleave primarily in the presence of GlcN6P [17]. This cleavage has been shown to destabilize *glmS* mRNA and thus to down-regulate *glmS* expression [18]. Interestingly, biochemical study revealed that *glmS* ribozyme is not an allosteric ribozyme *per se*, since GlcN6P does not allosterically regulate *glmS* ribozyme but rather serves as a cofactor which directly contributes to catalysis [19].

More recently, the engineering of artificial riboswitches based on *cis*-cleaving aptazymes has achieved some success. By connecting the anti-theophylline or anti-tetracycline aptamers to the tobacco ringspot virus (TRSV) HHRz via rationally designed or selected communication modules, Win and Smolke engineered aptazymes that, when inserted to the 3′ UTR of the GFP gene, could regulate GFP expression in yeast in response to theophylline or tetracycline concentration [14]. The reported dynamic range of GFP expression level was 20∼25-fold (Figure 2 of [14]). However, closer inspection of the raw data provided in the supplementary material (Figure S13 of [14]) showed that the dynamic range of GFP expression level was actually much lower. Among all the aptazyme constructs that were designed and tested, most displayed only ∼1.5-fold regulation and the best ones displayed ∼2.5-fold regulation. The discrepancy between the interpretation and the data was due to redefinition of the word ‘fold’ by the authors. Although the word ‘fold’ is generally used to express the ratio of two quantities, Win and Smolke used ‘fold’ as a unit of absolute quantity of GFP expression [14]. For example, the GFP expression level from an unengineered plasmid was defined as ‘50 fold.’ Therefore, when the GFP expression level from an engineered plasmid changed from ‘20 fold’ in the absence of theophylline to ‘43 fold’ in the presence of theophylline, a dynamic range of ‘(43−20 = ) 23 fold’ could be claimed. Most researchers would instead estimate the dynamic range to be (43/20 = ) 2.2-fold. Win and Smolke have also reported that multiple aptazymes inserted into the 3′ UTR could act as logic gates for gene expression, but the raw data necessary to evaluate these claims were not immediately available [15].

These designs were of necessity eukaryote-specific, since the 3′ polyA:5′ cap interaction is crucial for efficient protein translation. A prokaryote-specific system has been developed by Wieland et al. in which the ribosome-binding site (RBS) of a reporter gene was embedded in stem I of the Schistosomal HHRz, such that the self-cleavage of the HHRz liberated the RBS for translation initiation [12],[13]. Through rational design and genetic screening, a theophylline-responsive aptazyme that exhibited 10-fold regulation of the expression of the reporter gene was generated. The fold-regulation achieved by these authors (1.2- to 10- fold) are far smaller than those that have been routinely demonstrated *in vitro* (10^2^-∼10^4^- fold ).

To explain this discrepancy, we will explore a simple kinetic model. In this model, the eukaryotic-specific system, where an aptazyme is placed within the 3′ UTR of a mRNA, will be used. That said, it should be noted that self-cleaving HHRzs placed within the 5′ UTR can abet even stronger inhibition of gene expression [20], but such a model would be inherently more challenging because it would have to take into account the continuous scanning by the pre-initiation complex.

### Modeling inhibition of gene expression by a constitutively active ribozyme

**Figure 3:**
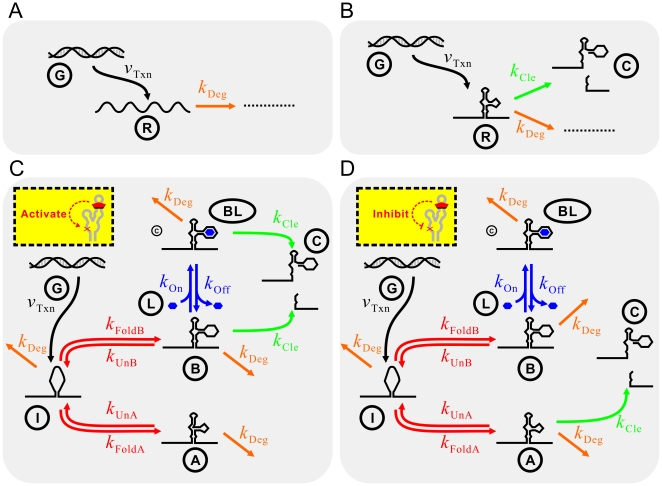
Models of aptazyme-based riboswitches. (A) Kinetic model for mRNA metabolism in the absence of ribozyme or aptazyme cleavage. (B) Kinetic model for mRNA metabolism when a constitutively active ribozyme is inserted into the 3′ UTR. (C) Kinetic model for mRNA metabolism when a ligand-activated aptazyme is inserted into the 3′ UTR. (D) Kinetic model for mRNA metabolism when a ligand-inhibited aptazyme is inserted into the 3′ UTR.

We first model how gene expression can be inhibited by a constitutively active, self-cleaving ribozyme (**Figures 3A and 3B**). In these models, we assume that the steady-state concentration of a protein is proportional to the steady state concentration of its intact mRNA. In contrast, mRNA with a cleaved 3′ UTR is assumed to have a negligible translation efficiency or is rapidly degraded [21].

In the absence of ribozyme cleavage (**Figure 3A**) the steady state concentration of mRNA ([*R*]_ss_) is 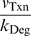. When a constitutively active self-cleaving ribozyme is inserted to the 3′ UTR of the mRNA (**Figure 3B**), the steady state concentration of intact mRNA should depend on its cleavage rate, as well as on the transcription and degradation rates, specifically:
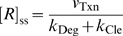
(17)


If we define the relative steady-state intact mRNA concentration without ribozyme as 1, then the relative steady-state intact mRNA concentration of an mRNA that harbors a ribozyme is:
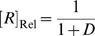
(18)where 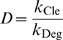 is the ratio of cleavage rate constant to the spontaneous degradation rate constant. The extent to which gene expression can be inhibited by an inserted ribozyme is directly determined by this ratio *D*, which implies that the rate of spontaneous degradation of mRNA also directly influences how much inhibition a given ribozyme can potentially achieve [22].

### Modeling regulation of gene expression by ligand-activated aptazymes

As before, we assume that the inactive conformer in a two-state model is completely inactive, and that the active conformer has the same cleavage rate constant as the ribozyme sans aptamer. The kinetic model for gene regulation via ligand-activated self-cleavage is shown in **Figure 3C**. For simplicity only the 3′ UTR is shown. In this model, mRNA is transcribed from the ‘gene’ (*G*) with a zero-order rate constant of *v*_Txn_. The nascent transcript (*I*) can fold into either aptazyme conformer [cleavage-incompetent conformer (*A*) or cleavage-competent conformer (*B*)] with folding and unfolding rate constants *k*_FoldA_, *k*_FoldB_ and *k*_UnA_, *k*_UnB_, respectively. The *B* (but not *A*) conformer can also bind the ligand *L* to form aptazyme∶ligand complex *BL* which has the same catalytic activity as *B* (*k*_Cle_). The second-order association rate constant and first-order dissociation rate constant are denoted as *k*_On_ and *k*_Off_, respectively.

Under this model (see **Text S2** for derivation), the relationship between steady-state relative concentration of intact mRNA (including *I*, *A*, *B* and *BL*) and the concentration of total ligand *L* ([*L*_tot_]) is expressed in the following equation:
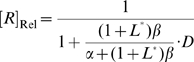
(19)where
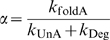
(20)

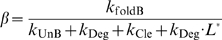
(21)and
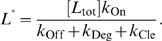
(22)


This definition of relative concentration 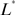 is similar to our earlier definition of relative ligand concentration, except that in this case *K*_d_ is replaced by:
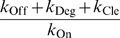
which we term the apparent dissociation constant and denote as 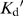. 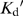 is similar in form to *K*_d_ (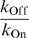) and will have a similar value to *K*_d_ when the dissociation rate constant of the ligand∶aptamer complex (*k*_Off_ values typically 10^−3^ to 10^1^ s^−1^) is much higher than the cleavage rate constant of the ribozyme (*k*_Cle_ values typically 10^−2^ to 1 s^−1^). However, it may also have a larger value than *K*_d_ when *k*_Off_ is comparable to or lower than *k*_Cle_. Again, 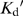 is the scaling factor for ligand concentration.

Since the degradation rate constant of mRNA in eukaryotic cells is much slower (by up to 10 orders of magnitude; [23]) than structural transition, ligand dissociation, and ribozyme cleavage rates, *α* and *β* should have values similar to the equilibrium constants for the reactions *I*↔*A* (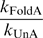) and *I*↔*B* (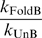). Notably, *β* can be treated as a constant although it is actually a function of ligand concentration.

When *β* is treated as a constant, 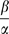 is similar to *K*_int_ in **Figure 2** and consequently 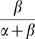 is equivalent to the cleavage tendency *ω*. Moreover, since the folded state is typically of lower energy (and thus more occupied) than the intermediate (*I*) or unfolded state, *α* is usually much greater than 1. Given these two conditions, the equation (19) can be written as:
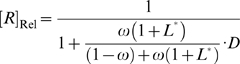
(23)


It is interesting that equation (23) can be simply obtained by replacing *k*_Cle_ in (17) with *k*_app_ in (4). This suggests that the equation for the function of aptazymes *in vitro* (4) can be used for aptazymes *in vivo*, with the only significant error coming when *k*_Cle_ is on the same order as or larger than *k*_Off_, which would in turn lead to a significant difference between 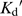 and *K*_d_.

### Design principles for ligand-activated aptazymes as *in vivo* riboswitches

#### Characteristics of the transfer function

The aptazyme regulation of steady-state mRNA concentration can be thought of as ‘cascaded’ hyperbolic control in which the apparent cleavage activity of the ribozyme (*k*_app_) is hyperbolically controlled by relative ligand concentration (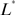) and [*R*]_Rel_ is in turn hyperbolically controlled by the cleavage activity of the ribozyme. Mathematically it can be proven that regulatory elements that exhibit hyperbolic responsivity also exhibit hyperbolic responsivity when coupled in series. In general, if:
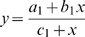
and
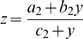
then:
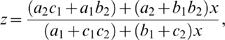
which means *z* is hyperbolically controlled by *x*.

**Figure 4:**
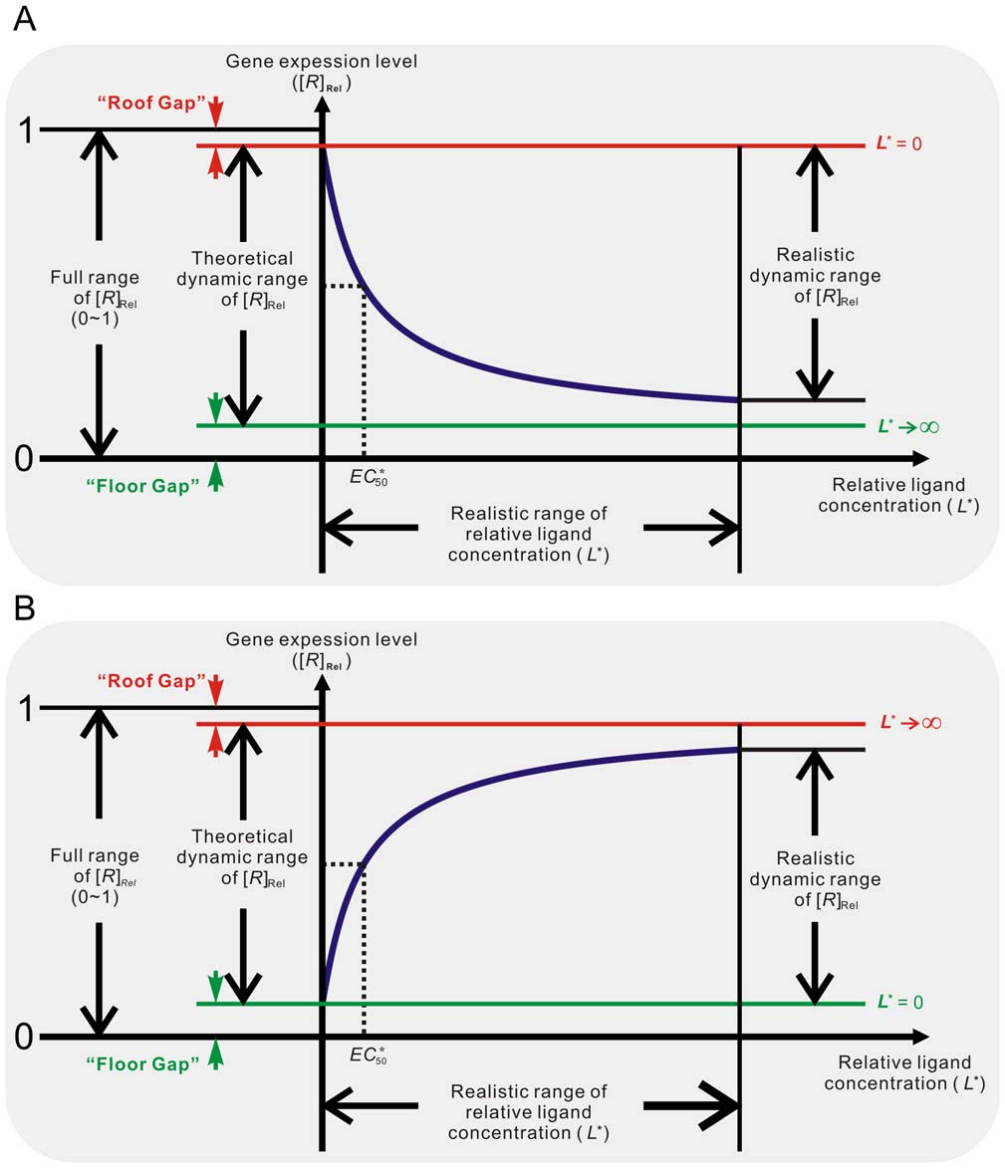
Performance of aptazymes as riboswitches. (A) Ligand-activated aptazyme; (B) Ligand-inhibited aptazyme. The relative ligand concentration (ligand concentration divided by the 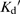 of the aptamer) and the relative gene expression level (steady-state mRNA concentration of the aptazyme-harboring gene divided by that of the un-engineered gene) are shown on the horizontal and vertical axes, respectively.

By applying this conclusion to equation (23) it can be seen that the [*R*]_Rel_-vs-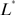 curve is a decreasing hyperbola (**Figure 4A**), whose descriptor can be obtained by rearranging (23) to:
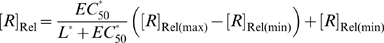
(24)where [*R*]_Rel(max)_ is the maximum value of [*R*]_Rel_ in the absence of ligand; [*R*]_Rel(min)_ is the lower limit of [*R*]_Rel_ and is approached at infinite ligand concentration; and 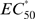 is the value of the dimensionless ligand concentration (obtained by dividing by 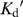) corresponding to the midpoint between [*R*]_Rel(min)_ and [*R*]_Rel(max)_. As a result:
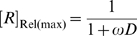
(25)

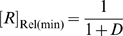
(26)

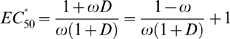
(27)


#### Limits on the theoretical dynamic range

The aim of the design process is to optimize both the dynamic range of gene expression (as shown by the range of [*R*]_Rel_) and sensitivity to effector (as shown by 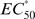). In order to have a large dynamic range of activity and modulation at low effector concentrations, aptamers, ribozymes, and mRNAs must be chosen that have optimal values of *K*_d_, *k*_Cle_, and *k*_Deg_, respectively. In addition, aptazyme cleavage tendency can be engineered to improve the dynamic range of activity and responsivity to effector.

For optimization of the dynamic range of activity, it is useful to think how closely the performance of the aptazyme can approach either completely cleaving or completely protecting a mRNA. The difference between complete cleavage of the mRNA and the theoretical minimum steady-state mRNA level that can be obtained in the presence of the aptazyme will be called the ‘Floor Gap’ (**Figure 4A**). The difference between complete protection and the theoretical maximum steady-state mRNA level in the presence of the aptazyme will be called the ‘Roof Gap’ (**Figure 4A**). The aptamer, ribozyme, mRNA, and aptazyme variables must be chosen so to have as narrow a ‘Roof Gap’ and ‘Floor Gap’ as possible, while still maintaining high ligand sensitivity (low 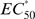).

Going by equation (26), it is clear that for a ligand-activated aptazyme the ‘Floor Gap’ is solely dependent on the intrinsic variable *D* (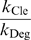). The ‘Floor Gap’ can only be narrowed by choosing or engineering faster ribozymes and/or more stable mRNAs.

In contrast, the ‘Roof Gap’ and 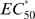 are dependent upon the cleavage tendency of the aptazyme. They also have additional limitations. According to (27), 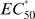 is always greater than 1 since *ω* is always smaller than 1. Thus 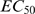 is always greater than 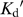. Moreover, by comparing equations (25) and (27) we can appreciate the relationship between the maximum amount of intact mRNA at steady-state and 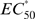. Under the common conditions *D*≫1 and *ω* is very small (see below):
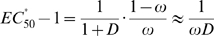
and thus
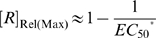
(28)or
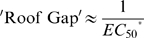
Therefore, ‘Roof Gap’ is inversely proportional to 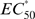, which is in turn determined by *D* and the cleavage tendency.

An additional criterion that can be used to evaluate the system is the fold-inhibition that occurs over the theoretical dynamic range of gene expression, denoted as 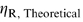:
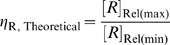



The ‘Roof Gap’ can be narrowed by engineering a very small cleavage tendency, i.e., by heavily disfavoring the cleavage-competent conformer (albeit at the cost of a rising 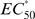). Under these circumstances, the primary determinant of 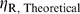 will be the ‘Floor Gap’, or [*R*]_Rel(min)_. For instance, when *D* can be made to be as high as 1000, a riboswitch with a theoretical ∼1000-fold inhibition (assuming no constraints on ligand concentration) can be engineered by designing the cleavage tendency to be ca. 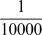. Given these parameters 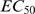 would be around 10 times *K*_d_′. Practically, for stringent regulation (>10-fold), we believe that *D* should be at least 10. This condition (high *D*, low cleavage tendency) satisfies equation (28), and is equivalent to saying that a high [*R*]_Rel(max)_ requires a high 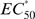. However, (28) also shows that when 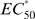 is greater than ∼5, further increases of 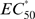 produce only marginal improvements in [*R*]_Rel(max)_.

This analysis suggests that the principles that apply *in vivo* are drastically different from those that apply to *in vitro* biosensors, primarily because the observed signals are different from one another (in one case, a direct readout of catalysis, in the other, a readout ‘buffered’ by transcription and degradation). From equation (9) we can see that in the *in vitro* case where *k*_app_ is essentially the observed signal a 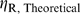 of 1000 would therefore require an 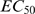 of 1,000 times *K*_d_. In contrast, for the *in vivo* case, when observing [*R*]_Rel_ the same 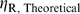 can be obtained with higher ligand sensitivity (i.e., with an 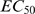 of ca. only 10 times *K*_d_′, as detailed above).

#### Limits on the realistic dynamic range

**Figure 5:**
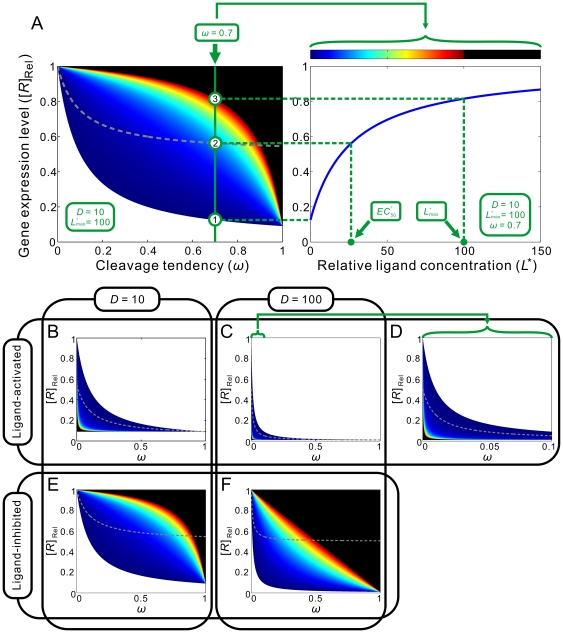
Regulatory landscapes for aptazyme-based riboswitches. (A) A guide to interpreting the regulatory landscape figures. When the type of aptazyme (ligand-activated or ligand-inhibited) and the values of *D* and 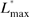 are all known, the regulatory landscape of a given riboswitch can be determined. The left panel shows an example of a ligand-inhibited aptazyme with 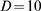 and 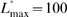. Different cleavage tendencies (*ω*, horizontal axis) and relative gene expression levels (vertical axis) are related by relative ligand concentrations (indicated by color mapping; the color scale is shown on the top of the right panel). The dynamic range of activities for a given *ω* can be determined by drawing a vertical line and looking at the relative gene expression levels at relative ligand concentrations 0 and 100. Such a vertical line is shown for a *ω* of 0.7, and the dynamic range (the ratio of the values at points 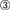 and 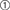) is ca. 6-fold. The relative concentration at point 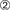 corresponds to the 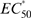 of this riboswitch. The dashed gray line represents the span of relative *EC*_50_ (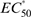) values. (B to D) The regulatory landscapes of ligand-activated aptazymes with different *D* values ( = 10 and 100) but the same 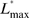 value ( = 100). Note that panel D is an expanded view of panel C where *ω* varies from 0 to 0.1 (instead of 0 to 1). (E to F) The regulatory landscapes of ligand-inhibited aptazymes with different *D* values ( = 10 and 100) but the same 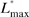 value ( = 100).

Although the compromise between ligand sensitivity and the theoretical dynamic range of activity *in vivo* is not as severe as was the case for the ligand-activated aptazyme *in vitro*, the [*R*]_Rel_-vs-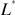 curve (in contrast to *k*_app_-vs-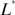 curve) is a decreasing hyperbola, and its lower limit is difficult to reach (see **Figure 4A**). Therefore when *D* is sufficiently large, the realistic dynamic range usually depends primarily on the maximum 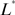. For the example above where the theoretical dynamic range of [*R*]_Rel_ is 1,000-fold, even a 500-fold reduction of [*R*]_Rel_ requires the intracellular ligand concentration to be at least 10,000×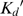, e.g. for an aptamer with a *K*_d_ of 100nM, the intracellular ligand concentration must be 1mM! The theoretical and realistic dynamic range of [*R*]_Rel_ as functions of cleavage tendency can be seen in the ‘regulatory landscape’ (**Figures 5** **)**. In this **Figure**, the relationship between three variables (cleavage tendency, [*R*]_Rel_, and ligand concentration) are plotted in two-dimensions. In order to achieve the third dimension, ligand concentration is colored. We also examine the relationships between these variables at two different values of *D*, 10 and 100.

In these plots the upper limit of achievable ligand concentration was arbitrarily chosen to be 100 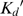. The theoretical dynamic range of [*R*]_Rel_ is encompassed within the colored (including black) region. The black areas represent those regions that are inaccessible due to difficult-to-achieve relative ligand concentrations. The 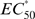 value is shown as a dashed line.

Based on the analyses above and an examination of **Figure 5**, we can qualitatively conclude that the primary variables that limit the performance of a ligand-activated aptazyme as a gene-regulatory element are *D* and 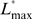. Therefore, in optimizing riboswitches based on ligand-activated aptazymes one must: (i) attempt to achieve the tightest ligand-binding possible; (ii) use or engineer a faster ribozyme and/or a more stable mRNA; (iii) appropriately disfavor the cleavage-competent conformation; and (iv) choose a ligand with high cell-permeability and low cytotoxicity.

More quantitatively, the optimal cleavage tendency *ω* can be determined when the limiting factors *D* and 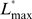 are both known. By defining 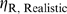 as the fold-inhibition yielded by the aptazyme over the realistic dynamic range of [*R*]_Rel_ (or formally: 
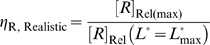
), the relationship between 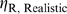 and *ω* can be analytically obtained:
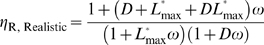
(29)


**Figure 6:**
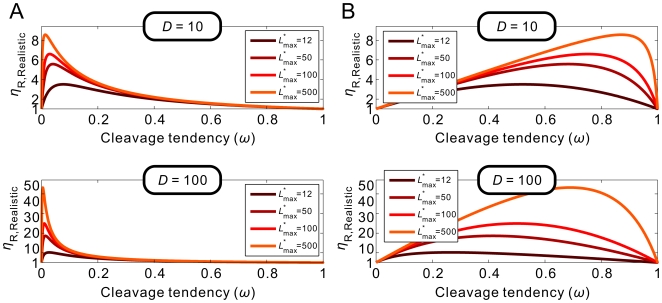
Quantitative relationships between 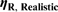 and cleavage tendency ***ω***. For ligand-activated aptazymes (A) and ligand-inhibited aptazymes (B), 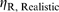 is defined as fold-inhibition and fold-activation of gene expression across the realistic dynamic range of gene expression. The relationship between 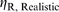 and cleavage tendency *ω* is shown for different, maximum possible relative ligand concentrations (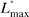; shown in different colors).

As shown in **Figure 6A**, for any given *D* and 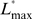 there are always *ω* ‘sweet spots’ where 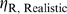 is maximized. Around these ‘sweet spots’ 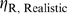 is highly sensitive to *ω*, especially when *D* and 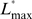 are high. The position of the *ω* ‘sweet spot’ (optimal *ω*) can be analytically obtained by solving 
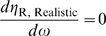
. However, this analytical result does not elucidate mechanistic understanding and is thus not shown.

### Modeling regulation of gene expression by ligand-inhibited aptazymes

The model for a ligand-inhibited self-cleaving ribozyme is diagramed in **Figure 3D**. The primary difference from the model for a ligand-activated aptazyme (**Figure 3C**) is that now only the conformer *A*, instead of both *B* and *BL*, can undergo self-cleavage. Given the parameters in **Figure 3C**, the relationship between relative steady-state concentration of intact mRNA ([*R*]_Rel_) and ligand concentration ([*L*_tot_]) is (see **Text S2** for derivation):
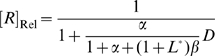
(30)where:
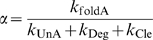
(31)

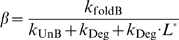
(32)

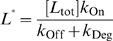
(33)


In this case the apparent dissociation constant 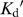 (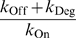) is closer in value to *K*_d_ since *k*_Cle_ does not appear in the definition of 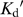. As before, *α* and *β* are similar to the equilibrium constants for the reactions *I*↔*A* (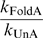) and *I*↔*B* (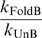), respectively, and *β* can be treated as a constant. Given that 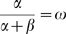, when *α* is much greater than 1 then equation (30) can be re-written as:
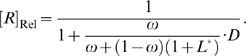
(34)


Since the inhibition of aptazyme cleavage would result in a increase of gene expression, the [*R*]_Rel_-vs-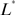 curve is an increasing hyperbola, whose descriptor can be obtained by re-writing (34) to:
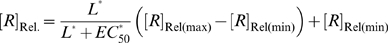
where:
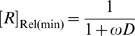
(35)

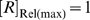
(36)and
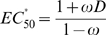
(37)


From these results it can be seen that the ‘Roof Gap’ (**Figure 4B**) for a ligand-inhibited aptazyme is always 0, since the mRNA can theoretically be completely protected when the concentration of the ligand approaches infinite. In contrast, the width of the ‘Floor Gap’ is dependent on *D* and the cleavage tendency *ω*. As before, the theoretical and realistic dynamic ranges of gene expression are graphically represented as a regulatory landscape (**Figures 5E–5F**). Analytically, by defining:
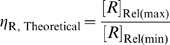
and
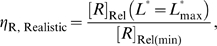
it can be shown that:
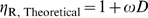
(38)and
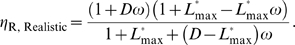
(39)


Once again, for each given *D* and 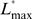 there is an optimal *ω* to maximize 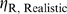 (**Figure 6B**). Interestingly, though, for ligand-inhibited aptazymes a much wider range of cleavage tendencies give satisfactory 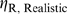 values (**Figures 6B∼6D**).

### Analytical import of the model

A major advance in our modeling compared to previous work ([16]) is that we provide practical guidelines for what experiments should be carried out to develop a quantitative understanding and prediction of riboswitch function. Based on our analysis, the performance of an aptazyme-based riboswitch can be quantitatively predicted when four parameters are known: (i) the gene expression level of an unengineered mRNA; (ii) the ratio of the ribozyme cleavage rate constant to the mRNA degradation rate constant (*D*); (iii) cleavage tendency of the aptazyme (*ω*); and (iv) the maximum available relative concentration of ligand (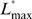).

Among these four parameters, the gene expression level of an unengineered mRNA can be trivially measured. Using equation (18), *D* can be obtained by measuring the gene expression level of an mRNA harboring a ribozyme sans aptamer at its 3′ UTR (or elsewhere). Once *D* is determined, the cleavage tendency can be predicted based on RNA folding energetics or by measuring the gene expression level of an aptazyme-harboring mRNA in the absence of ligand, according to equations (25) and (35).

The only parameter that cannot be directly measured is 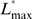. However, 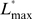 is ligand-specific, aptamer-specific, and organism-specific, but not design-specific. Therefore if the 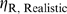 for one aptazyme is measured, 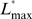 can be calculated and used to predict the performance of other aptazymes which contain the same aptamer and are used in the same organism. To calculate 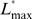 from 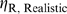 one need only solve equations (29) and (39), yielding:
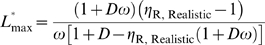
(40)for ligand-activated aptazymes and
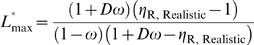
(41)for ligand-inhibited aptazymes.

With such a theoretical framework we can attempt not only to promulgate engineering principles, but also to analyze previously designed aptazyme-based riboswitches. As we discussed above, Win and Smolke generated a series of theophylline-responsive hammerhead ribozymes by grafting the anti-theophylline aptamer onto loop I or loop II of the TRSV ribozyme via various communication domains [14]. When these different constructs were placed in the 3′ UTR of a reporter gene (GFP) modest ∼2-fold effects on gene regulation were observed. One rationale for the disappointing results was that introduction of aptamer domains into loop I and loop II disrupted a known, critical tertiary interaction [24]. Although the original TRSV ribozyme inserted into the 3′ UTR can inhibit the expression of GFP expression to 2% of the unengineered mRNA level, when loop II was extended the inhibition was only to ∼10%. If the steady-state GFP signal reflects the steady-state concentration of intact mRNA, the *D* value for the engineered aptazymes was thus likely to be ∼10. Therefore, the maximum activation and inhibition could never exceed 10-fold, as shown by **Figures 6A and 6B** **(top panels)**. The constructs were inherently restricted by their very design.

Beyond limitations on catalysis, we also suspect that there were limitations on either the allosteric binding sites or the available intracellular ligand concentration. Using the data from **Figure S13** of Win and Smolke [14] and equations (25) and (35), the cleavage tendencies of each aptazyme were calculated (**Table 1**). 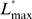 was also calculated from each aptazyme construct using equations (40) and (41) (**Table 1**). Although many 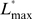 values fall into a narrow range, they were not consistent. Possible explanations for this inconsistency include: (i) the existence of ‘non-productive’ aptazyme conformations not considered in the model (e.g., a non-binding and non-cleaving conformation of the ligand-inhibited aptazyme); and (ii) the possibility that the basic functionality of either the aptamer or the ribozyme were significantly altered in the aptazyme designs.

**Table 1:** Analysis of the experimental data in Win and Smolke (2007) [14].

Construct	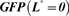 (a.u.†)	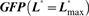 (a.u.†)	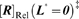	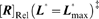	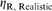	*ω* ≠	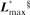
L2bulgeOff1	31	14	0.62	0.28	2.2	0.06	4.3
L2bulgeOff2	15	8	0.3	0.16	1.9	0.23	2.6
L2bulgeOff3	10	7	0.2	0.14	1.4	0.40	1.4
L1cm10	27	13	0.54	0.26	2.1	0.09	3.3
L2cm1	36	27	0.72	0.54	1.3	0.04	1.3
L2cm4	39	20	0.78	0.4	2.0	0.03	5.1
L2cm5	42	28	0.84	0.56	1.5	0.02	3.4
L2cm9	16	6	0.32	0.12	2.7	0.21	9.2*
L2cmd	22	7	0.44	0.14	3.1	0.13	9.9*
L2bulge1	20	43	0.4	0.86	2.2	0.15	9.7*
L2bulge2	22	38	0.44	0.76	1.7	0.13	3.5
L2bulge3	25	34	0.5	0.68	1.4	0.10	1.3
L2bulge4	21	41	0.42	0.82	2.0	0.14	6.1
L2bulge5	41	49	0.82	0.98	1.2	0.02	10.0*
L2bulge8	6	18	0.12	0.36	3.0	0.73	11.7*
L2bulge9	15	36	0.3	0.72	2.4	0.23	6.5

**†:** This arbitrary unit (a.u.) for GFP expression is defined by Win and Smolke [14]. With this definition, the GFP expression level for unengineered mRNA is 50 a.u.. Source of data: SI Figure 13 of Win and Smolke [14].

**‡:** According to the definition of [*R*]_Rel_ (18) and the assumption that gene expression level is proportional to steady-state intact mRNA level, 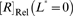 and 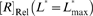 are calculated with the following equations:
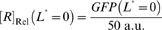


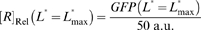
.

**≠:** The cleavage tendencies (*ω*) are calculated with equations (25) and (35) for ligand-activated and ligand-inhibited aptazymes, respectively. The value of *D* used in these calculations is 10 (see text).

**§:** The maximum available relative ligand concentrations (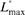) are calculated with equations (40) and (41) for ligand-activated aptazymes and ligand-inhibited aptazymes, respectively.

*: The five constructs that showed high 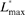 are denoted with ^*^.

To further our analysis, we assume that the aptazymes showing the largest 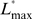 (∼12) did not operate under the caveats stated above. If so, the maximum available cellular theophylline concentration was only about 12 times the 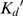 of the anti-theophylline aptamer. The anti-theophylline aptamer has a reported *K*_d_<1µM [25]. Assuming the aptamer retains its affinity for theophylline in the cellular environment, the calculated 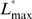 indicates that the intracellular concentration would be on the order of 12 µM, even though the extracellular concentration of theophylline was 5mM. This discrepancy is consistent with an early finding that the intracellular concentration of theophylline in *E.coli* is 10^3^-fold lower than the concentration in media [26], and with the previous performance of an engineered antiswitch in yeast [8].

**Figure 7:**
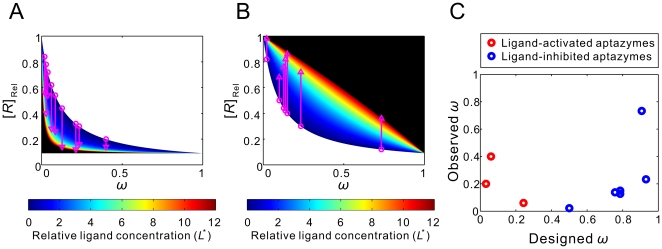
Analysis of an aptazyme-based riboswitch *in vivo* (Win and Smolke (2007) [14]). The regulatory landscapes for ligand-activated (A) or ligand-inhibited (B) aptazyme-based riboswitches are shown. In these landscapes the value of *D* is 10, and the value of 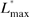 is 12. The published relative expression levels of the designed aptazymes in the absence of ligand (pink circles) and at saturating concentrations of ligand (pink triangles) are plotted versus the calculated cleavage tendency [using equations (25) and (35)]. (C) The discrepancy between designed cleavage tendencies (see Methods) and observed cleavage tendencies (**Table 1**).

The comparison between the model and the experimental data from these studies can be visualized in the regulatory landscapes shown in **Figure 7A and 7B**, where the calculated cleavage tendencies and the relative gene expression values are shown both in the absence of theophylline (circles) and in the presence of 5 mM theophylline (triangles). For most constructs, there was quantitative agreement between the model and experimental data with acceptable variance. It should be noted that if we had used the original, published estimates for the fold-change due to the aptazyme there would have been virtually no agreement between model and experiment.

With aptazymes that had an intrinsically limited *D* (∼10) and a small upper limit of *L* ^*^ (∼12), it was ultimately to be expected that the maximum fold-change that might be available through optimization of the communication module was only ∼3.5-fold (**Figures 6A and 6B** **, upper panels**) for both ligand-activated and ligand-inhibited aptazymes.

In order to actually obtain better aptazyme and riboswitch functionality both a larger *D* and a higher upper limit of *L* ^*^ must be engineered. Our model predicts that by using a 10-fold more stable mRNA the maximum fold-change can be increased to ∼7-fold (**Figure 6A and 6B** **, lower panels**; keeping the upper limit of *L* ^*^ constant). For this more stable mRNA when *L* ^*^ is also increased to 50 (by using a tighter binding aptamer∶ligand pair and/or a ligand that is better able to penetrate the cell), ∼17-fold regulation can be achieved (**Figure 6A and 6B** **, lower panels**).

In summary, the dynamic range of gene expression in the current aptazyme-based riboswitch system is severely limited by the cleavage rate of the ribozyme relative to spontaneous mRNA degradation rate and the achievable intracellular ligand concentration relative to the *in vivo K*_d_ of the aptamer. Reasonable improvements of these factors should lead to a wider dynamic range of gene expression.

### Challenges and future directions

Although throughout the above analyses we assume that the cleavage tendency can be freely tuned, this is based on the assumption that for a given sequence design the aptazyme conformations and their relative energetics can be reliably predicted. This assumption is questionable. For example, we have recently designed a series of biosensors based on the anti-thrombin aptamer, and demonstrated that biosensor properties did not align with the stabilities based on secondary structural features alone, but were fit much better by measured stabilities [27]. Similarly, attempts to computationally design hammerhead aptazymes based only on secondary structural hypotheses (the ‘slip structure’ model; [9]) yielded aptazymes that were much less activated [28].

Such discrepancies are likely to be even greater when intracellular energetics need to be predicted. For example, for the aptazymes designed by Win and Smolke [14], the cleavage tendencies calculated from experimental data (**Table 1**) largely disagree with the predicted cleavage tendencies calculated from the thermodynamics data (taken from Table S1 of [14]), as shown in **Figure 7C**. In principle, designed aptazymes should be characterized *in vitro* to better understand whether and how they fit either *in silico* data or the *in vivo* data. Similarly, a recent attempt at model-driven design of allosteric shRNAs also yielded only qualitative agreement with modeling based on secondary structures [29].

To better ensure coherence between model and reality, many assumptions and predictions made in our model of aptazyme-based biosensors and riboswitches need to be tested experimentally. First of all, it is critical to test to what extent the two-state structural and energetic model is acceptable. In a recent elegant study on the kinetics of a previously engineered theophylline-activated hammerhead ribozyme [9], de Silva and Walter observed four conformations relevant to activation using single-molecule fluorescent resonance energy transfer (FRET) [30]. Moreover, upon the addition of theophylline the conformational change of the aptamer domain was observed to be much faster than that of the ribozyme core. Based on these results the authors suggested a model for ligand-induced conformational change in which the aptamer domain is capable of binding ligand even in the cleavage-incompetent conformation of the aptazyme. Consequently, the ligand binding of the aptamer domain primes the conformational change of the communication domain and the ribozyme domain (induced fit). Whether this mechanism proves to be general will strongly impact how the kinetics of effector modulation are modeled, and may alter the equilibrium arguments we make herein, depending on how the different energy states are populated.

In addition, parameters relevant to the *in vivo* environment need to be characterized in greater detail in order to understand aptazyme function. For example, translation efficiency and the half-life of cleaved mRNAs should be carefully determined since these factors, although ignored in the current model, would contribute to the background expression level when a ribozyme or aptazyme is cleaving at full speed [21]. A more fundamental and largely unknown issue is how the energetics and kinetics of RNA folding are influenced by the biochemical properties (ionic strength, viscosity, the presence of RNA chaperons and helicases) in cellular environments. While predictive models are incomplete in the absence of such information, it is nonetheless worthwhile to formulate them so that the functionality of aptazymes can be more routinely evaluated as these additional variables are acquired.

## Methods

The derivations of the fundamental equations (equations (2), (3), (19) and (30)) that describe how energetic parameters dictate the performance of aptazymes *in vitro* and *in vivo* are provided in the **Text S1 and S2**. All figures were produced with MatLab using the equations described in the text.

The Supplemental Information and Figure 13 from Win and Smolke [14] were used to derive data for our analyses. The ‘designed cleavage tendency’ presented in our **Figure 7C** was calculated using the equations:
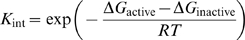
and
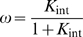
where the values of 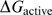 and 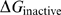 were taken from the Supplemental Information Table 2 of reference [14].

## Supporting Information

Text S1 Derivation of equations
(0.03 MB DOC)

Text S2 Derivation of equations
(0.11 MB DOC)

Text S3 Summary of terms
(0.07 MB DOC)
