# Supplementary material for: Design Principles for Ligand-Sensing, Conformation-Switching Ribozymes
Source: PLoS Comput Biol. 2009 Dec 24;5(12):e1000620. doi: 10.1371/journal.pcbi.1000620 (PMC2789328; doi:10.1371/journal.pcbi.1000620)
Supplement: Text S1 — Derivation of equations (0.03 MB DOC) [file pcbi.1000620.s001.doc]

**Text S1**: Derivation of the transfer function for aptazymes as *in vitro* biosensors

The definition of *K*_int_ and *K*_a(B)_ is:

$\frac{[B]}{[A]}=K_{\mathrm{int}}$ **(S.1)**
and:

$\frac{[BL]}{[L][B]}=K_{\text{a(B)}}=\frac{1}{K_{\text{d(B)}}}$. **(S.2)**

Since in this analysis we ignore the presence of *AL* (see main text), only the dissociation constant of the complex of the *B* conformer with the ligand (*K*_d(B)_) affects the behavior of the aptazyme. Therefore in the following derivation we use the term ‘*K*_d_’ in place of ‘*K*_d(B)_’ for simplicity.

If we assume that the concentration of ligand is much greater than the concentration of aptazyme, the concentration of free ligand should be similar to the concentration of total ligand, and therefore:

$\frac{[BL]}{[B]}=\frac{[L_{\text{tot}}]}{K_{\text{d}}}$ **(S.3)**where [*L*_tot_] is the total concentration of the ligand.

It can be seen that for a given internal equilibrium constant *K*_int_ partitioning between *A*, *B*, and *BL* is determined by the ratio of [*L*_tot_] to *K*_d_. We define this ratio as the ‘relative ligand concentration,’ or *L*^*^ ($L^{*}=\frac{[L_{\text{tot}}]}{K_{\text{d}}}$). Therefore **(S.3)** can be rewritten as:

$\frac{[BL]}{[B]}=L^{*}$. (**S.4)**

From **(S.1)** and **(S.4)** the fraction of *A* can be deduced to be:

$f_{A}=\frac{[A]}{[A]+[B]+[BL]}=\frac{[A]}{[A]+K_{\text{int}}[A]+K_{\text{int}}L^{*}[A]}=\frac{1}{1+K_{\text{int}}+K_{\text{int}}L^{*}}$
and the total fractions of *B* and *BL* are:

$f_{B+BL}=\frac{[B]+[BL]}{[A]+[B]+[BL]}=\frac{K_{\text{int}}+K_{\text{int}}L^{*}}{1+K_{\text{int}}+K_{\text{int}}L^{*}}$.
