# Supplementary material for: Design Principles for Ligand-Sensing, Conformation-Switching Ribozymes
Source: PLoS Comput Biol. 2009 Dec 24;5(12):e1000620. doi: 10.1371/journal.pcbi.1000620 (PMC2789328; doi:10.1371/journal.pcbi.1000620)
Supplement: Text S2 — Derivation of equations (0.11 MB DOC) [file pcbi.1000620.s002.doc]

**Text S2**: Derivation of the transfer function for aptazymes as *in vivo* riboswitches

**Ligand-activated aptazymes***.*

Assuming that the kinetic parameters outlined in **Figure 3C** and the initial concentrations of each species are known, the concentration of each species at any time point can be obtained by integrating the differential equation set:

$\frac{d[I]}{dt}=v_{\text{Txn}}-[I](k_{\text{FoldA}}+k_{\text{FoldB}}+k_{\text{Deg}})$ **(S.5)**

$\frac{d[A]}{dt}=[I]k_{\text{FoldA}}-[A](k_{\text{UnA}}+k_{\text{Deg}})$ **(S.6)**

$\frac{d[B]}{dt}=[I]k_{\text{foldB}}+[BL]k_{\text{Off}}-[B](k_{\text{UnB}}+k_{\text{Deg}}+k_{\text{Cle}}+k_{\text{On}}[L])$ **(S.7)**

$\frac{d[BL]}{dt}=[B][L]k_{\text{On}}-[BL](k_{\text{Off}}+k_{\text{Deg}}+k_{\text{Cle}})$ **(S.8)**

The steady-state concentrations of each species can be solved by setting equations **(S.5)**~**(S.8)** to zero. Then, to obtain insights into how the concentrations of different species behave as a function of ligand concentration we can determine how intact mRNA is partitioned amongst the conformations *I*, *A*, *B* and *BL*.

First we consider the steady-state ratio of *B* to *BL*, which is determined by the concentration of free ligand [*L*]. We assume that the total ligand concentration is much greater than the concentration of RNA, and therefore that free ligand concentration [*L*] approximates total ligand concentration [*L*_tot_]. Based on **(S.8)** at steady-state:

$[B][L_{\text{tot}}]k_{\text{On}}=[BL](k_{\text{Off}}+k_{\text{Deg}}+k_{\text{Cle}})$
and thus:

$\frac{[BL]}{[B][L_{\text{tot}}]}=\frac{k_{\text{On}}}{k_{\text{Off}}+k_{\text{Deg}}+k_{\text{Cle}}}$. **(S.9)**

The ratio $\frac{k_{\text{Off}}+k_{\text{Deg}}+k_{\text{Cle}}}{k_{\text{On}}}$ has a form similar to that of the dissociation constant, *K*_d_. Thus we term this ratio as $K_{\text{d}}'$. Formally:

$K_{\text{d}}'=\frac{k_{\text{Off}}+k_{\text{Deg}}+k_{\text{Cle}}}{k_{\text{On}}}$ **(S.10)**

We can divide *L*_tot_ by$K_{\text{d}}'$ to obtain a dimensionless measure of ligand concentration, $L^{*}$. This allows **(S.9)** to be written as:

$$\frac{[BL]}{[B]}=L^{*}$$

We next consider the ratio of [*A*] to [*I*], and the ratio of [*B*] to [*I*] at steady-state. By letting $\frac{d[A]}{dt}=0$ and $\frac{d[B]}{dt}=0$in **(S.6)** and (**S.7)** we find:

$$\frac{[A]}{[I]}=\frac{k_{\text{foldA}}}{k_{\text{UnA}}+k_{\text{Cle}}}$$

$$\frac{[B]}{[I]}=\frac{k_{\text{foldB}}}{k_{\text{UnB}}+k_{\text{Deg}}+k_{\text{Cle}}+k_{\text{Deg}}\cdot L^{*}}$$

For simplicity, we define the new parameters:

$\alpha=\frac{k_{\text{foldA}}}{k_{\text{UnA}}+k_{\text{Deg}}}$ **(S.11)**

$\beta=\frac{k_{\text{foldB}}}{k_{\text{UnB}}+k_{\text{Deg}}+k_{\text{Cle}}+k_{\text{Deg}}\cdot L^{*}}$ **(S.12)**

where *α* and *β* have similar meanings and values to the equilibrium constants for the reactions *I*↔*A* ($\frac{k_{\text{FoldA}}}{k_{\text{UnA}}}$) and *I*↔*B* ($\frac{k_{\text{FoldB}}}{k_{\text{UnB}}}$), respectively, but are further affected by the degradation rate constant *k*_Deg_, the cleavage rate constant *k*_Cle_, and the dimensionless ligand concentration $L^{*}$. It should be noted that although *β* is a function of $L^{*}$, it can be viewed as a constant in most cases since the degradation rate constants for most eukaryotic mRNAs are much smaller (by up to 10 orders of magnitude; Al-Hashimi and Walter, 2008) than the rate constants for conformational change.

Therefore, the ratio of species *A*, *B*, and *BL* to *I* can be written as:

$\frac{[A]}{[I]}=\alpha$, $\frac{[B]}{[I]}=\beta$, $\frac{[BL]}{[I]}=\frac{[BL]}{[B]}\frac{[B]}{[I]}=L^{*}\cdot\beta$

If we express the concentration of all intact mRNAs as [*R*], we then have:

$$\frac{[R]}{[I]}=\frac{[I]+[A]+[B]+[C]}{[I]}=1+\alpha+(1+L^{*})\beta$$

and

$$\frac{[I]}{[R]}=\frac{1}{1+\alpha+(1+L^{*})\beta}$$

$$\frac{[A]}{[R]}=\frac{\alpha}{1+\alpha+(1+L^{*})\beta}$$

$$\frac{[B]}{[R]}=\frac{\beta}{1+\alpha+(1+L^{*})\beta}$$

$$\frac{[BL]}{[R]}=\frac{L^{*}\cdot\beta}{1+\alpha+(1+L^{*})\beta}$$

Since all intact mRNA species have the same degradation rate, while only *B* and *BL* decay through aptazyme cleavage, we can further derive:

$$[R]k_{\text{Deg}}+([B]+[C])k_{\text{Cle}}=v_{\text{Txn}}$$

$$[R]\left(k_{\text{Deg}}+\frac{[B]+[C]}{[R]}k_{\text{Cle}}\right)=v_{\text{Txn}}$$

$$[R]\left(k_{\text{Deg}}+\frac{(1+L^{*})\beta}{1+\alpha+(1+L^{*})\beta}k_{\text{Cle}}\right)=v_{\text{Txn}}$$

$$[R]=\frac{v_{\text{Txn}}}{k_{\text{Deg}}+\frac{(1+L^{*})\beta}{1+\alpha+(1+L^{*})\beta}k_{\text{Cle}}}$$

If we again define the steady-state intact mRNA concentration in the absence of aptazyme as 1 (as in **(18)**), then the relative intact mRNA concentration in the presence of aptazyme can be written as:

$$[R]_{\text{Rel}}=\frac{[R]}{\frac{v_{\text{Txn}}}{k_{\text{Deg}}}}=\frac{1}{1+\frac{(1+L^{*})\beta}{1+\alpha+(1+L^{*})\beta}\cdot D}$$

**Ligand-inhibited aptazymes.**

The model for a ligand-inhibited, self-cleaving ribozyme is diagramed in **Figure 3D**. The primary difference between this and the model for a ligand-activated aptazyme (**Figure 3C**) is that now only the conformer *A*, rather than both *B* and *BL*, can undergo self-cleavage. At steady state,

$$[B][L_{\text{tot}}]k_{\text{On}}=[BL](k_{\text{Off}}+k_{\text{Deg}})$$

and,

$\frac{[BL]}{[B][L_{\text{tot}}]}=\frac{k_{\text{On}}}{k_{\text{Off}}+k_{\text{Deg}}}$.

Similarly we define the apparent dissociation constant $K_{\text{d}}'$ for ligand-inhibited aptazyme as:

$K_{\text{d}}'=\frac{k_{\text{Off}}+k_{\text{Deg}}}{k_{\text{On}}}$ **(S.13)**

We then focus on the ratios of $\frac{[A]}{[I]}$ (*α*) and $\frac{[A]}{[I]}$ (*β*):

$\alpha=\frac{[A]}{[I]}=\frac{k_{\text{foldA}}}{k_{\text{UnA}}+k_{\text{Deg}}+k_{\text{Cle}}}$ **(S.14)**

$\beta=\frac{[B]}{[I]}=\frac{k_{\text{foldB}}}{k_{\text{UnB}}+k_{\text{Deg}}+k_{\text{Deg}}\cdot L^{*}}$ **(S.15)**

As before, *β* is a function of $L^{*}$, but now can be treated as a constant (again assuming that the structural transition happens much faster than cleavage and degradation). The fraction of cleavage-competent conformer thereby becomes:

$$f_{\text{cc}}=\frac{[A]}{[I]+[A]+[B]+[BL]}=\frac{\alpha}{1+\alpha+(1+L^{*})\beta}\approx\frac{\alpha}{\alpha+(1+L^{*})\beta}$$

and therefore:

$$[R]_{\text{Rel}}=\frac{1}{1+\frac{\alpha}{\alpha+(1+L^{*})\beta}D}$$
