# Supplementary material for: Design Principles for Ligand-Sensing, Conformation-Switching Ribozymes
Source: PLoS Comput Biol. 2009 Dec 24;5(12):e1000620. doi: 10.1371/journal.pcbi.1000620 (PMC2789328; doi:10.1371/journal.pcbi.1000620)
Supplement: Text S3 — Summary of terms (0.07 MB DOC) [file pcbi.1000620.s003.doc]

**Text S3**: Summary of terms

**General terms:**

- *K*_int_: *K*_int_ is the equilibrium constant between the two conformers of the aptazyme in the absence of ligand.
- *ω*: Cleavage tendency *ω* is the fraction of cleavage-competent conformer in the absence of ligand.

**Terms in the context of aptazymes as in vitro biosensors:**

- *k*_app_: *k*_app_ is the apparent cleavage rate constant when a self-cleaving aptazyme is tested *in vitro* under certain concentration of ligand.
- $L^{*}$: Relative ligand concentration $L^{*}$ is the ratio of total ligand concentration to the *K*_d_ of the aptamer domain to its ligand, or in equation, $L^{*}$ = [*L*_tot_]/*K*_d_.
- $EC_{50}$: $EC_{50}$ is the concentration of total ligand at which the aptazyme is half-activated or half-inhibited.
- $EC_{50}^{*}$: $EC_{50}^{*}$ is the relative ligand concentration at which the aptazyme is half-activated or half-inhibited, or in equation, $EC_{50}^{*}$= $EC_{50}$/*K*_d_. The relationships between $EC_{50}^{*}$ and *ω* for ligand-activated aptazymes and ligand-inhibited aptazymes are shown in equations (8) and (14), respectively.
- $L_{\max}^{*}$: $L_{\max}^{*}$ is the highest possible relative ligand concentration, or in other words, the highest possible ligand concentration divided by *K*_d_ of the aptamer domain to its ligand.
- $\eta_{\text{R, Theoretical}}$: For ligand-activated aptazymes, $\eta_{\text{R, Theoretical}}$ is the fold-increase of *k*_app_ when ligand concentration increases from 0 to infinite. The relationship between $\eta_{\text{R, Theoretical}}$ and *ω* is shown in equation (9). $\eta_{\text{R, Theoretical}}$ is not defined for ligand-inhibited aptazymes.
- $\eta_{\text{R, Realistic}}$: $\eta_{\text{R, Realistic}}$ is the fold-increase or fold-decrease of *k*_app_ when ligand concentration increases from 0 to the highest possible ligand concentration for ligand-activated aptazymes and ligand-inhibited aptazymes, respectively. The dependences of $\eta_{\text{R, Realistic}}$ on $L_{\max}^{*}$ and *ω* for ligand-activated aptazymes and ligand-inhibited aptazymes are shown in equations (11) and (16), respectively.

**Terms in the context of aptazymes as in vivo riboswitches:**

- $[R]_{\text{Rel}}$: Relative mRNA level $[R]_{\text{Rel}}$ is the steady-state concentration of mRNA that harbors an aptazyme or ribozyme at its 3′ UTR divided by the steady-state concentration of the same mRNA without an aptazyme or ribozyme. Thus when the cleavage activity of the aptazyme or ribozyme is zero, $[R]_{\text{Rel}}$ = 1; when the activity of the aptazyme or ribozyme is infinitely high, $[R]_{\text{Rel}}$ = 0.
- *D*: *D* is the ratio of the cleavage rate constant of a constitutively active ribozyme to the spontaneous degradation rate constant of the mRNA.
- *α*: *α* is the ratio of the concentration of conformer *A* to the concentration of conformer *I* (in Figure 3C and 3D) at steady state (see equations **S.11** and **S.14**).
- *β*: *β*  is the ratio of the concentration of conformer *B* to the concentration of conformer *I* (in Figure 3C and 3D) at steady state (see equations **S.12** and **S.15**).
- $K_{\text{d}}'$: $K_{\text{d}}'$ is the apparent *in vivo* dissociation rate constant of the aptamer domain to its ligand in the presence of mRNA degradation and aptazyme cleavage. The definitions of $K_{\text{d}}'$ for ligand-activated and ligand-inhibited aptazyme are shown in equations (**S.10** and **S.13**). $K_{\text{d}}'$ usually has similar value to the true *K*_d_ of the aptamer domain to its ligand.
- $L^{*}$: Relative ligand concentration $L^{*}$ is the ratio of total ligand concentration to the $K_{\text{d}}'$ of the aptazyme, or in equation, $L^{*}$=[*L*_tot_]/$K_{\text{d}}'$.
- $EC_{50}$: $EC_{50}$ is the concentration of total ligand at which the relative mRNA level $[R]_{\text{Rel}}$ is in the middle of the theoretical dynamic range of $[R]_{\text{Rel}}$.
- $EC_{50}^{*}$: $EC_{50}^{*}$ is the relative concentration of total ligand at which the relative mRNA level $[R]_{\text{Rel}}$ is in the middle of the theoretical dynamic range of $[R]_{\text{Rel}}$, or in equation, $EC_{50}^{*}$=$EC_{50}$/$K_{\text{d}}'$. The dependences of $EC_{50}^{*}$ on *ω* and *D* for ligand-activated aptazymes and ligand-inhibited aptazymes are shown in equations (27) and (37), respectively.
- $L_{\max}^{*}$: $L_{\max}^{*}$ is the highest possible relative ligand concentration, or in other words, the highest possible ligand concentration divided by $K_{\text{d}}'$.
- $\eta_{\text{R, Theoretical}}$: $\eta_{\text{R, Theoretical}}$ is the fold-increase or fold-decrease of $[R]_{\text{Rel}}$ when ligand concentration increases from 0 to infinite for ligand-inhibited aptazymes and ligand-activated aptazymes, respectively.
- $\eta_{\text{R, Realistic}}$: $\eta_{\text{R, Realistic}}$ is the fold-increase or fold-decrease of $[R]_{\text{Rel}}$ when ligand concentration increases from 0 to the highest possible ligand concentration for ligand-inhibited aptazymes and ligand-activated aptazymes, respectively. The dependences of $\eta_{\text{R, Realistic}}$ on $L_{\max}^{*}$, *ω* and *D* for ligand-activated aptazymes and ligand-inhibited aptazymes are shown in equations (29) and (39), respectively.
